# Sphingomyelin Deacylase, the Enzyme Involved in the Pathogenesis of Atopic Dermatitis, Is Identical to the β-Subunit of Acid Ceramidase

**DOI:** 10.3390/ijms21228789

**Published:** 2020-11-20

**Authors:** Yasuhiro Teranishi, Hiroshi Kuwahara, Masaru Ueda, Tadashi Takemura, Masanori Kusumoto, Keiji Nakamura, Jun Sakai, Toru Kimura, Yasuji Furutani, Makoto Kawashima, Genji Imokawa, Mari Nogami-Itoh

**Affiliations:** 1 Drug Research Division, Dainippon Sumitomo Pharma Co., Ltd., Osaka City, Osaka 554-0022, Japan; yasuhiro-teranishi@ds-pharma.co.jp (Y.T.); kuwaharah@sc.sumitomo-chem.co.jp (H.K.); masaruueda824@gmail.com (M.U.); tadashi-takemura@ds-pharma.co.jp (T.T.); kusumotom@sc.sumitomo-chem.co.jp (M.K.); kashibanara@yahoo.co.jp (K.N.); bacwe400@gmail.com (J.S.); toru-kimura@ds-pharma.co.jp (T.K.); yasuji-furutani@ds-pharma.co.jp (Y.F.); 2 Department of Dermatology, Tokyo Women’s Medical University, Tokyo 162-8666, Japan; mkawashima@tokyowomen-u.ac.jp; 3 Center for Bioscience Research & Education, Utsunomiya University, 350 Mine Utsunomiya, Tochigi 321-8505, Japan; 4 National Institutes of Biomedical Innovation, Health and Nutrition, AI Center for Health and Biomedical Research 7-6-8 Asagi Saito Ibaraki-city, Osaka 567-0085, Japan

**Keywords:** atopic dermatitis, ceramide, ceramide deficiency, barrier function, water reservoir faction, stratum corneum, sphingomyelin deacylase, sphingosylphosphorylcholine, acid ceramidase

## Abstract

A ceramide deficiency in the stratum corneum (SC) is an essential etiologic factor for the dry and barrier-disrupted skin of patients with atopic dermatitis (AD). Previously, we reported that sphingomyelin (SM) deacylase, which hydrolyzes SM and glucosylceramide at the acyl site to yield their lysoforms sphingosylphosphorylcholine (SPC) and glucosylsphingosine, respectively, instead of ceramide and/or acylceramide, is over-expressed in AD skin and results in a ceramide deficiency. Although the enzymatic properties of SM deacylase have been clarified, the enzyme itself remains unidentified. In this study, we purified and characterized SM deacylase from rat skin. The activities of SM deacylase and acid ceramidase (aCDase) were measured using SM and ceramide as substrates by tandem mass spectrometry by monitoring the production of SPC and sphingosine, respectively. Levels of SM deacylase activity from various rat organs were higher in the order of skin > lung > heart. By successive chromatography using Phenyl-5PW, Rotofor, SP-Sepharose, Superdex 200 and Shodex RP18-415, SM deacylase was purified to homogeneity with a single band of an apparent molecular mass of 43 kDa with an enrichment of > 14,000-fold. Analysis by MALDI-TOF MS/MS using a protein spot with SM deacylase activity separated by 2D-SDS-PAGE allowed its amino acid sequence to be determined and identified as the β-subunit of aCDase, which consists of α- and β-subunits linked by amino bonds and a single S-S bond. Western blotting of samples treated with 2-mercaptoethanol revealed that, whereas recombinant human aCDase was recognized by antibodies to the α-subunit at ~56 kDa and ~13 kDa and the β-subunit at ~43 kDa, the purified SM deacylase was detectable only by the antibody to the β-subunit at ~43 kDa. Breaking the S-S bond of recombinant human aCDase with dithiothreitol elicited the activity of SM deacylase with ~40 kDa upon gel chromatography. These results provide new insights into the essential role of SM deacylase expressed as an aCDase-degrading β-subunit that evokes the ceramide deficiency in AD skin.

## 1. Introduction

Atopic dermatitis (AD) is characterized pathophysiologically by accentuated cutaneous permeability [1] and deficient water reservoir functions even in the stratum corneum (SC) of non-lesional skin [2]. These dysfunctions of the SC are essential etiological factors that elicit recurrent dermatitis with a high susceptibility to irritants and/or allergens as well as atopic dry skin which is a prerequisite factor for the easily provoked itching. Accumulating evidence has indicated that the barrier-disrupted dry skin of AD patients is mainly associated with significantly decreased levels of total ceramides in the SC [3,4,5,6,7,8,9,10]. This pathophysiological association is based on evidence that ceramide acts as a water modulator by holding water molecules [11] and as a permeability barrier by forming multi-layered lamellar structures with other lipids between cells in the SC layers [12,13,14,15,16]. Further, the lipid lamellar organization in the SC of AD skin with perturbed barrier and water reservoir functions is distinctly altered as a result of changes in ceramide profiles including the ceramide composition and their alkyl chain lengths [17]. The essential role of the ceramide deficiency in the pathogenesis of AD is also corroborated by our recent study demonstrating that repetitive topical applications of a synthetic pseudo-ceramide (pCer) to AD skin significantly improved inflammation and atopic dry skin as well as the SC barrier/water reservoir function by switching the ceramide profile to a healthy skin phenotype [18]. Nevertheless, these clinical and functional improvements in the SC can be achieved without any recovery of the decreased levels of total endogenous ceramides but with applied and compensated pCer remaining at a similar level to existing endogenous ceramides. The remaining levels (µg/ng SC protein) of applied pCer in the SC of AD skin are well correlated (*n* = 39, *r* = 0.447, *p* = 0.005) with the increased water content measured by conductance, whereas any ceramide species at the level of µg/ng SC protein are not paralleled by the increased water content. This suggests that total ceramide levels, including penetrated pCer in the SC, are more essential in maintaining the barrier and water reservoir functions than are the differential ceramide profiles and play an essential role in improving clinical symptoms. Thus, it is intriguing to know what biological factors would trigger the epidermis to down-regulate the synthesis of the SC ceramides in the AD skin.

Ceramide levels in the SC are modulated by the balance of three enzymes involved in sphingolipid hydrolysis, β-glucocerebrosidase (BGCase), acid sphingomyelinase (aSMase) and acid ceramidase (aCDase), following the secretion of lamellar granules (LGs) in the interface between the stratum granulosum and the SC. Those three sphingolipid hydrolysis enzymes, except for aCDase, are not attenuated at the enzymatic activity level [6,19,20] or at the protein level [21,22] in the non-lesional epidermis or the non-lesional SC from AD skin, although one study did report a decreased activity of aSMase [23]. Further, other sphingolipid metabolic enzymes that function upstream of the hydrolysis of glucosylceramide (GCer) and sphingomyelin (SM) in the epidermis, such as serine-palmitoyl transferase (SPT), stearoyl CoA desaturase (SCD) [22], ceramide synthases (CERS) 1-5 [22], GCer synthase (GCERS), alkyl chain elongation enzymes [22] and SM synthase (SMS) have never been reported in uninflamed non-lesional AD skin to be implicated in the ceramide deficiency. 

As a causative etiologic biofactor that can elicit a ceramide deficiency even under these normal sphingolipid metabolic situations, we have discovered a novel enzyme, SM deacylase, which cleaves the N-acyl linkage of SM and GCer [2,6,19,24,25,26]. The activity of SM deacylase is significantly increased in the AD lesional epidermis as well as in the involved and uninvolved SC of AD skin, but not in the skin of patients with contact dermatitis or chronic eczema, compared with healthy controls (HCs) [6,25]. SM deacylase competes with aSMase and BGCase to hydrolyze their common substrates, SM or GCer, to yield their lysoforms sphingosylphosphorylcholine (SPC) or glucosylsphingosine (GS), respectively, instead of ceramide, that results in the ceramide deficiency in AD skin, independent of the activities of aSMase and BGCase. Consistently, those reaction products (SPC and GS) accumulate to a greater extent in the involved and uninvolved SC of AD skin compared with chronic eczema or contact dermatitis as well as HCs and, interestingly, the increased levels of SPC and GS are well correlated (*n* = 51, *r* = 0.44, *p* < 0.01 and *n* = 36, *r* = −0.41, *p* < 0.05) with the decreased levels of total ceramides or acylceramide (Cer[EOS]) (*n* = 52, *r* = −0.523, *p* < 0.0005), especially for GS [6,7]. It should be noted that the reported stimulatory effects of SPC on pigmentation by melanocytes [27] and on ICAM-1 expression [28] and keratinization (transglutaminase 1 expression) [29] by keratinocytes may contribute to the cutaneous clinical characteristics in atopic dry skin such as pigmented dirty skin [30], roughened and hardened SC cells and epidermis that is susceptible to allergens, respectively. Taken together, the above evidence suggests that two typical clinical features in atopic dry skin, water loss and barrier disruption, are associated with the ceramide deficiency that could be mediated via the elicitation and the enzymatic action of SM deacylase.

Thus, the specific expression of SM deacylase activity in AD skin provides a reasonable hypothesis to explain why the level of SC ceramide continues to be significantly down-regulated and is not up-regulated even by frequently induced inflammation in the SC of non-lesional AD skin [10] in concert with no substantial attenuation of the three major ceramide-related hydrolytic enzymes, the abnormalities of which markedly contrast with healthy skin. Therefore, it was important to purify the SM deacylase enzyme to homogeneity and to identify the detailed characteristics of this novel enzyme at the gene and protein levels. In the present study, we succeeded in purifying SM deacylase from rat skin to homogeneity and found that it is identical to the β-subunit of aCDase, which consists of α- and β-subunits linked by a single S–S bond, disruption of which by dithiothreitol results in eliciting the activity of SM deacylase.

## 2. Results

### 2.1. Tissue Distribution of SM Deacylase Activity 

**Figure 1:**
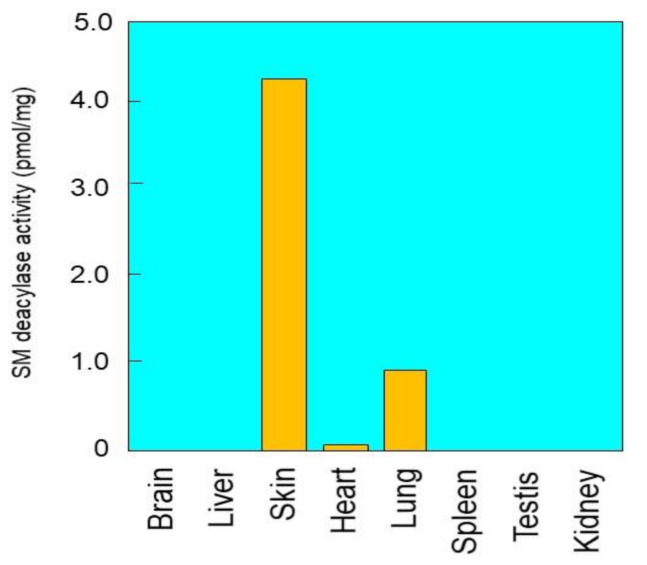
Distribution of sphingomyelin (SM) deacylase activity in rat tissues. SM deacylase solubilized by 1% n-octyl-β-d-glucoside from various rat tissues was incubated with 20 µM SM at 37 °C for 16 h. The reaction product (sphingosylphosphorylcholine, SPC) was quantified according to assay procedures as described in the Section 4.

To precisely measure SM deacylase activity, we developed a highly sensitive LC-MS/MS method, which has the capacity to measure the reaction product, SPC, over five orders of magnitude with an excellent linearity (R^2^ > 0.998). When SM deacylase activity was assayed in tissue extracts after 12,000× *g* centrifugation of homogenates of various rat organs, the highest activity was observed in the skin with a specific activity of 4.17 pmol/h/mg protein at 37 °C, followed by the lung and the heart (Figure 1). There was no detectable SM deacylase activity in the other organs tested. It should be noted that as for the presence of SM deacylase activity in healthy human skin, it was distinctly detectable, although to a lesser degree, in the SC or the epidermis of non-atopic healthy human skin [19,24]. 

### 2.2. Purification of SM Deacylase from Rat Skin

**Figure 2:**
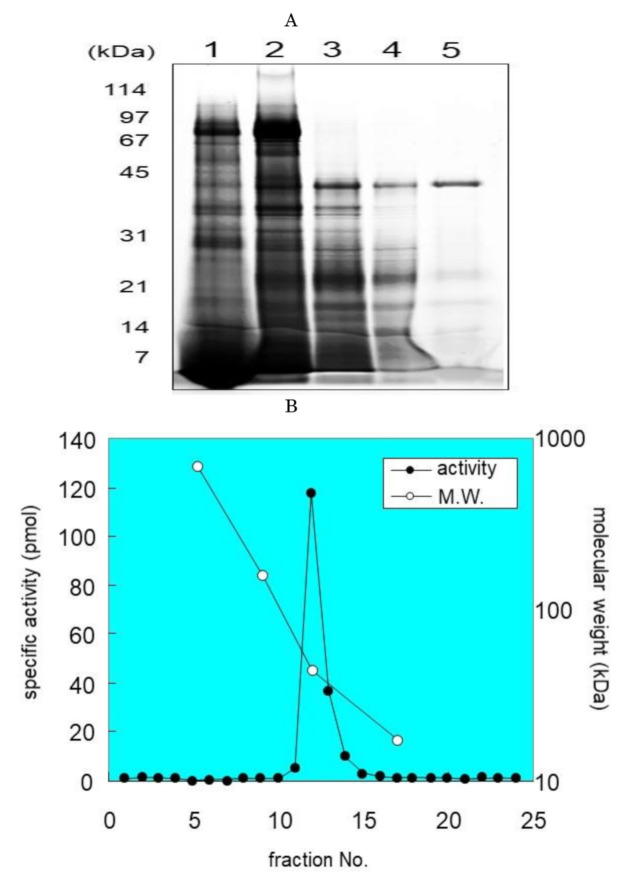

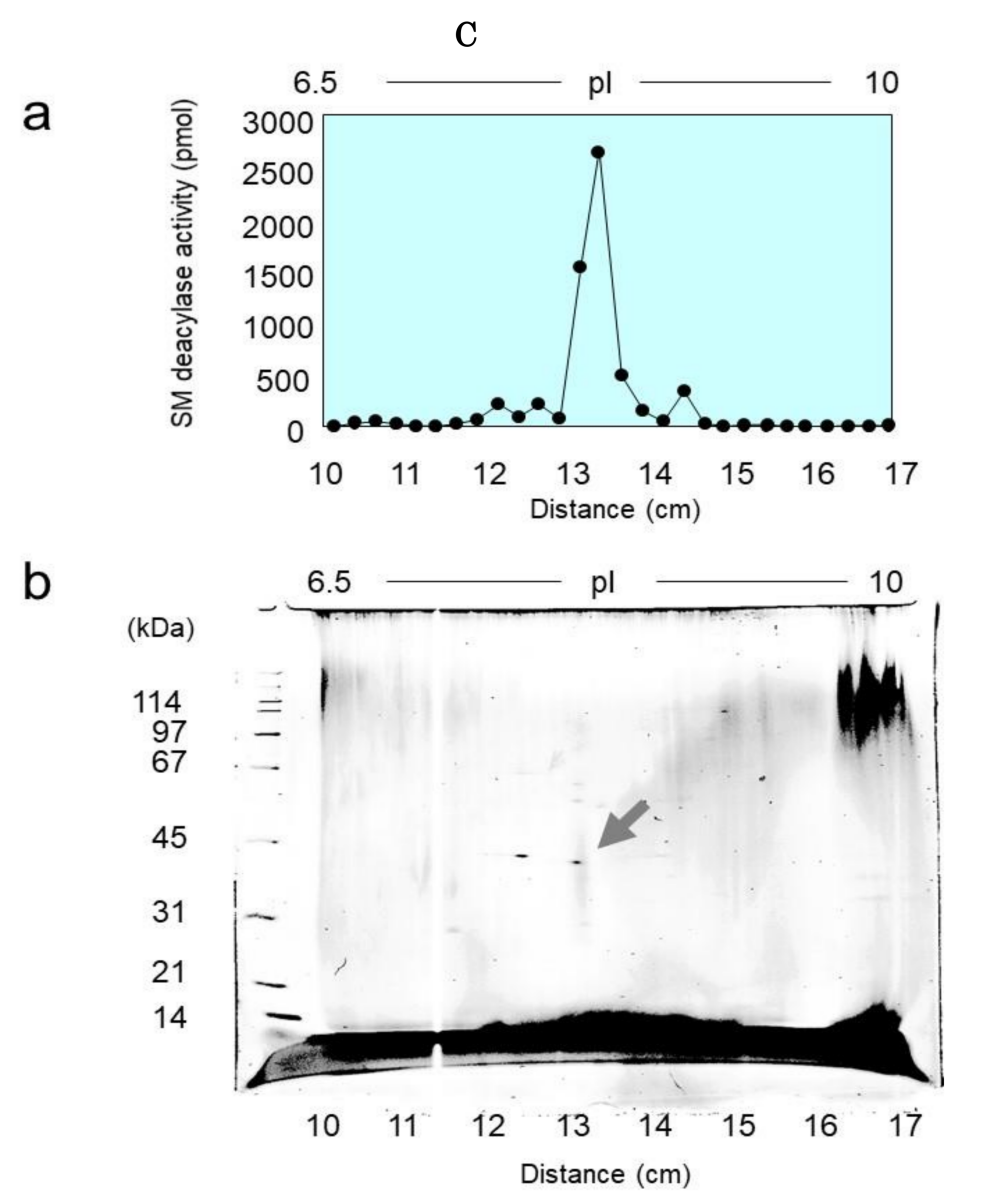
(**A**): SDS-PAGE of SM deacylase active fractions in each purification step. Aliquots of SM deacylase active fractions after each purification step were collected and analyzed by 12.5% SDS-PAGE, followed by staining with SYPRO Ruby and visualization of fluorescence using a Typhoon-9400 scanner. Lane 1, isoelectric focusing (Rotofor); Lane 2, ion exchange chromatography (SP Sepharose); Lane 3, first gel filtration chromatography (Superdex 200); Lane 4, second gel filtration chromatography (Superdex 200); Lane 5, affinity chromatography (RP-415 C20). (**B**): Estimation of the molecular weight of SM deacylase enzyme activity. SM deacylase purified from rat skin was separated by gel filtration chromatography using a Superdex 200 column. Eluted fractions were collected by volume and were assayed for SM deacylase activity (closed circles); estimated apparent molecular mass by protein standards (open circles); cytochrome C (124 kDa), albumin (66 kDa), ovalbumin (44 kDa), chymotrypsin (24 kDa). (**C**): Determination of SM deacylase active protein spot on 2D-PAGE gels. (**a**) Purified enzymes were isoelectrically focused at 12 W constant power for 4 h at 4 °C in native conditions. The IEF strips were cut into 40 equal parts and were then subjected to assays for SM deacylase activity. The activity in each slot was plotted along with the distance from the edge. (**b**) 2D electrophoresis was performed by mounting another IEF separated strip gel on top of an SDS-PAGE gel. After electrophoresis, the gel was stained by SYPRO Ruby and detected using a fluorescence image scanner. The pI range was from 6.5 to 10 and the molecular mass range was from 114 to 14 kDa, as indicated. The protein spot indicated by the arrow was prepared for MS/MS analysis. The two-dimensional gel image is representative of a minimum of three replicates.

For the purification of SM deacylase, we designed several purification steps. In brief, the homogenized rat skin was centrifuged at 12,000× *g* and the supernatant was subjected to ammonium sulfate precipitation. After re-solubilization, SM deacylase was purified to apparent homogeneity using a five-step procedure: hydrophobic chromatography, iso-electric focusing, ion-exchange chromatography, gel filtration chromatography and immune-affinity chromatography. Those procedures increased the specific enzyme activity approximately 14,000-fold, from 26 pmol/hr/mg to 363,919 pmol/h/mg protein (Table 1). The yield was 379% compared with the original homogenate. The yield of the IEF chromatography step was “4000”% and when all IEF-separated fractions were mixed, that high activity decreased to the original level, suggesting that this increase may be attributable to the removal of endogenous inhibitors of SM deacylase. The fractions containing SM deacylase activity in each purification step were analyzed by SDS-PAGE (Figure 2A) and, in the final step, the purified enzyme gave a major protein band around 42 kDa (Figure 2A). SM deacylase purified from rat skin was purified using a Superdex 200 column, and a plot of the elution of SM deacylase activity against a molecular mass standard curve revealed an apparent mass of approximately 40 kDa (Figure 2B), which agrees with the apparent molecular weight determined by SDS-PAGE. After purification of SM deacylase from rat skin using IEF, the separated strips were cut into 2.5 cm pieces and examined for SM deacylase activity, which had a peak at pI 8.0 (Figure 2C(a)). Those strips were also resolved by SDS-PAGE analysis and visualized by SYPRO Ruby staining (Figure 2C(b)). The molecular weight of the single spot obtained is in a good agreement with that determined by gel chromatography (Figure 2B) and isoelectric pI point of SM deacylase activity (Figure 2C(a,b)). 

### 2.3. Identification of Proteins with SM Deacylase Activity 

**Figure 3:**
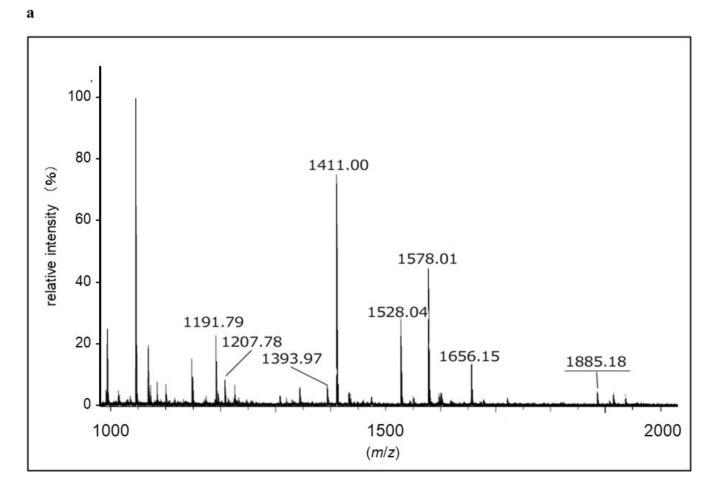

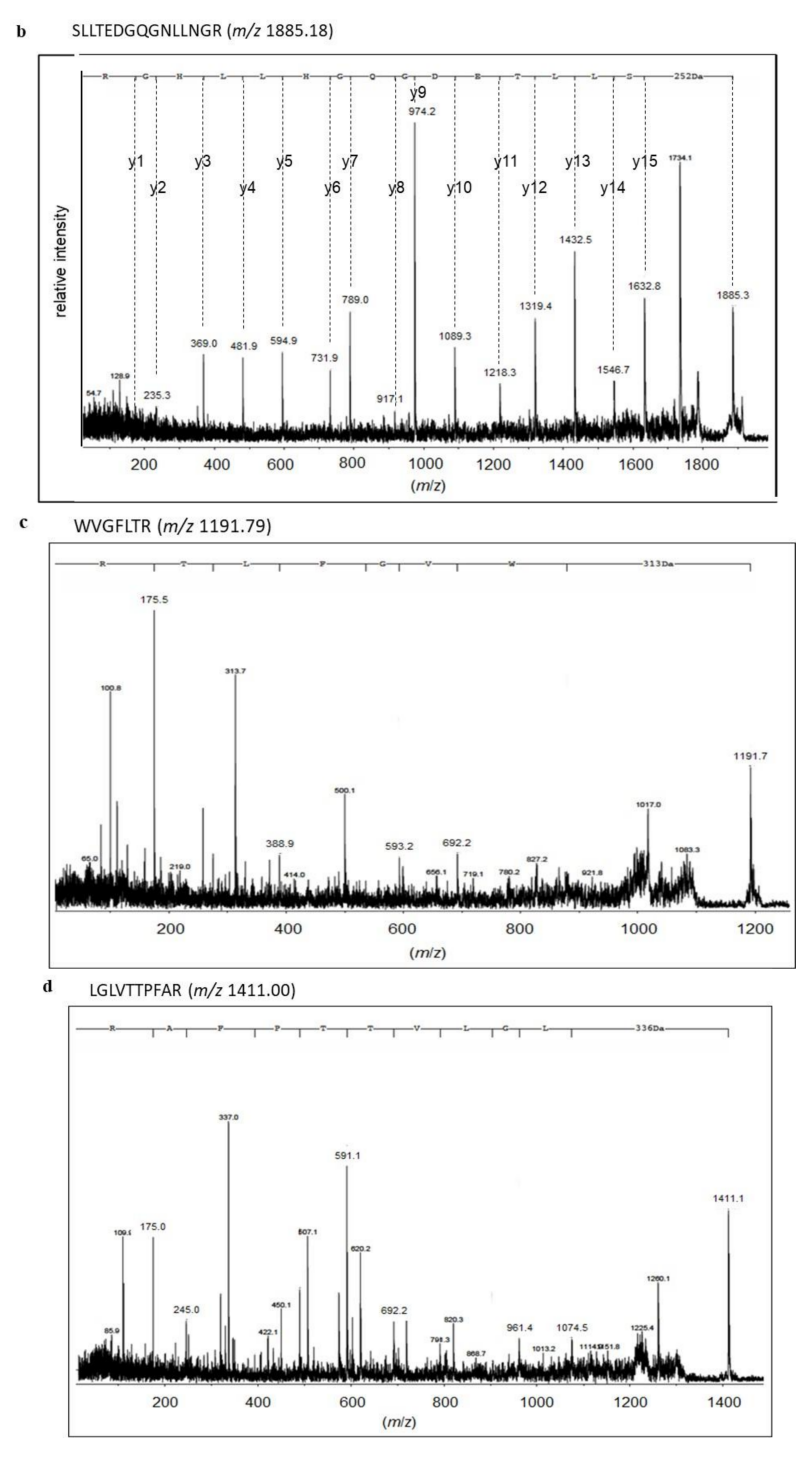

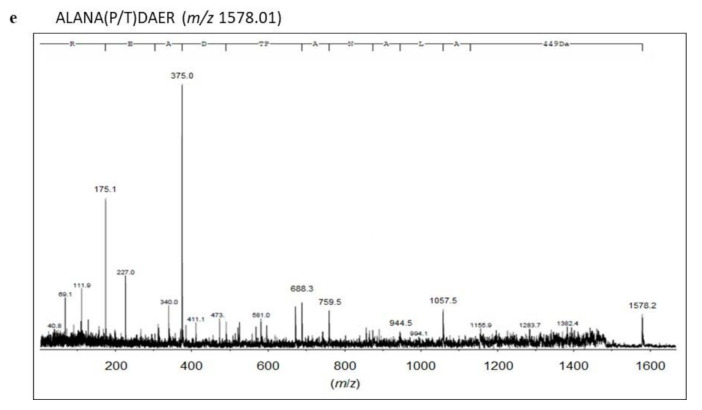
Identification of SM deacylase from rat skin. (**a**) MALDI-TOF-MS spectra from digested protein separated by 2D-PAGE; the x-axis represents the mass-to-charge ratio (*m*/*z*) whereas the y-axis represents relative abundance. (**b**–**e**) MS/MS spectrum of tryptic peptides from the SM deacylase active protein spot. Peptide sequences of ionic peaks obtained in (**a**) were analyzed according to their intensity and four peptide sequences were identified. Representative peptide fragmentation patterns are shown ((**b**): SLLTEDGQGNLLNGR, *m*/*z* 1885.18/(**c**): WVGFLTR (*m*/*z* 1191.7)/(**d**): LGLVTTPFAR (*m*/*z* 1411.0)/(**e**): ALANA(P/T)DAER (*m*/*z* 1578.0)) with y-series ions. The peptide (SLLTEDGQGNLLNGR) and another peptide (WVGFLTR) revealed a part of aCDase when referring to the MASCOT database.

**Figure 4:**
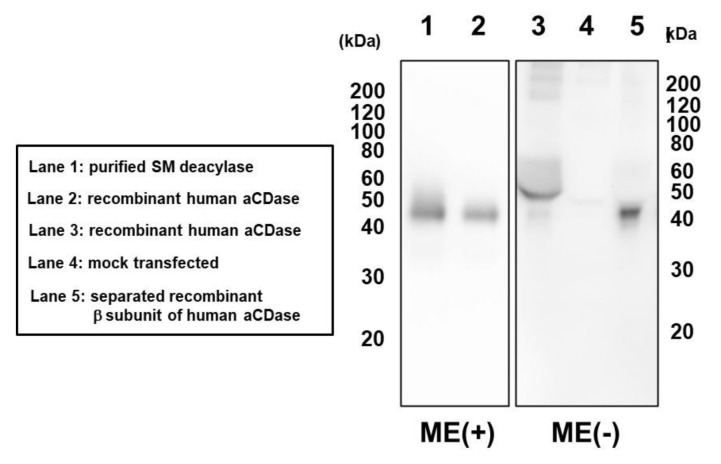
Subunit composition of purified SM deacylase recombinant human aCDase and separated recombinant β-subunit of human aCDase. The samples were separated by SDS-PAGE followed by immunoblot analysis using antibodies to the β-subunit (human) of aCDase. Before electrophoresis, samples were reduced with 5% 2-mercaptoethanol (ME) in the No 1 and 2 lines as shown by ME+ but not treated with ME in the No 3/4/5 lines, as shown by ME−. Lane 1, purified rat SM deacylase (ME+); Lane 2, recombinant human aCDase (ME+); Lane 3, recombinant human aCDase (ME−). Lane 4, mock transfected (ME−); Lane 5, separated recombinant β-subunit of human aCDase (ME−).

To identify protein spots with SM deacylase activity, we excised them from 2D-PAGE gels, and digested them with trypsin. Digested peptides were analyzed for their amino acid sequences using MALDI-TOF-MS. The peptide patterns detected by a TOF-MS analyzer are shown in Figure 3a. Among them, peaks with relatively high intensity were selected and analyzed for their sequence and four peptide fragments were successfully identified (Figure 3b–e). Using the MASCOT algorithm, two of the fragments were assigned to aCDase (Table 2). Since more than two peptides are necessary to identify a protein, this result indicates that this spot may certainly include aCDase [31,32]. As for the other identified peptides, their sequences were not included in the database. aCDase is a 43 kDa protein consisting of two subunits, a 13 kDa α-subunit and a 30 kDa β-subunit, and due to glycosylation, its apparent molecular weight is shifted to approximately 53 kDa [33,34,35]. The molecular weight of the identified protein spot is about 40 kDa (Figure 2C(b)) which is about 10 kDa smaller than the apparent molecular weight of mature aCDase determined by SDS-PAGE [33,34,35]. This result indicates that the SM deacylase purified from the rat skin may not be glycosylated or that its α-subunit is missing. The molecular weight of the purified SM deacylase was not affected by glycosidase treatment and we examined its subunit composition. Western blotting analysis using β-subunit specific antibodies under a reduced or non-reduced condition revealed that the SM deacylase purified from rat skin has a distinct band of approximately 40 kDa (lane 1) which is consistent with the band detected for recombinant human aCDase (lane 2) under the reduced conditions (ME+) (Figure 4). Under the non-reduced conditions (ME−), recombinant human aCDase had a distinct band of approximately 53 kDa of molecular weight (lane 3). Because we failed to express the recombinant β-subunit of human aCDase in the HEK293 cells transfected with expression vectors encoding the β-subunit of human aCDase, alternatively, we prepared the recombinant β-subunit of human aCDase after separation of the β-subunit from aCDase lacking an inter-subunit disulfide bond (C31A). The separated recombinant β-subunit of human aCDase demonstrated a distinct band of approximately 40 kDa of molecular weight in the non-reduced conditions (lane 5), which indicates a high quality of our prepared β-subunit-specific antibodies. Taken together, these results strongly suggest that the SM deacylase purified from rat skin is mainly composed of the β-subunit of aCDase. 

### 2.4. Treatment with DTT Separates SM Deacylase from Recombinant Human aCDase

**Figure 5:**
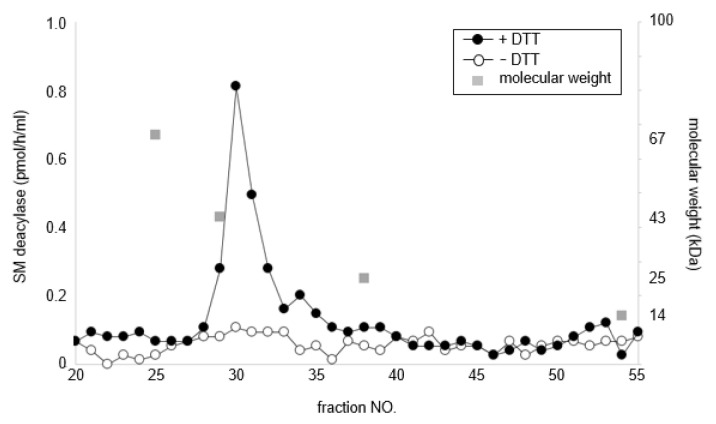
Dithiothreitol (DTT) separates SM deacylase from recombinant human aCDase. Recombinant human aCDase was incubated in a buffer containing 50 mM potassium acetate, pH 4.7 and 2.5% n-octyl-β-d-glucoside (*v*/*v*) for 60 min with (solid circle) or without (open circle) dithiothreitol (DTT) at 200 mM and was subjected to gel filtration chromatography using a Superdex 200 column. Proteins were eluted at a flow rate of 1 mL/min and fractions were collected by 0.25 mL. Aliquots of those fractions were analyzed for activities of SM deacylase.

To determine whether the β-subunit of human aCDase is identical to human SM deacylase, purified recombinant human aCDase was applied to Sephadex 200 gel chromatography before and after DTT treatment and each fraction was measured for the activity of SM deacylase. The results showed that while non-DTT-treated recombinant human aCDase had little SM deacylase activity, DTT treatment yielded SM deacylase-active fractions with a peak at approximately 40 kDa (Figure 5), which indicates that breaking the S-S bonds of recombinant human aCDase by dithiothreitol elicited the activity of SM deacylase. 

### 2.5. pH Dependence of SM Deacylase Activity

**Figure 6:**
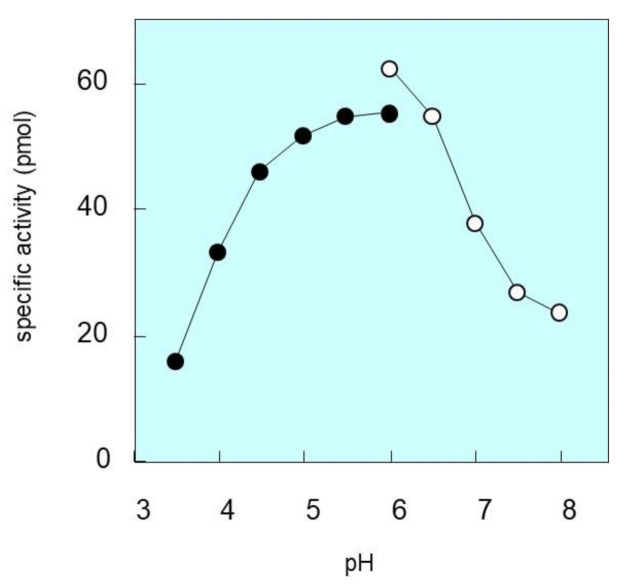
pH dependence of SM deacylase activity. SM deacylase activity of the purified and reduced enzyme was measured at the indicated pHs by examining the amount of SPC released. The pH value was adjusted using the following buffers: 50 mM potassium acetate (closed circles) or 50 mM sodium phosphate (open circles). In addition to those pH buffers, the enzyme reactions were performed by adding 20 mM CaCl_2_, 200 µM SM and 0.1% Triton X-100.

We then determined the pH dependency of purified SM deacylase. When the pH value was adjusted between 3.5 and 8.0, the SM deacylase activity was highest between 5.5 and 6.0 and was hardly detectable above pH 8.0 or below pH 3.5 (Figure 6). At pH 6.0, the activity was considerably higher in Tris-HCl than in citrate-sodium phosphate. This is a higher pH point than we previously reported [19], which again suggests the possibility that certain compounds or proteins that affect enzymatic characteristics were removed in the purification process. In this connection, we determined whether the α-subunit was removed during the IEF, but we could not detect that. Thus, at this time, it is not clear whether this change of specificity can be attributed to removal of the α-subunit or to the removal of some molecule other than the α-subunit during the purification process.

### 2.6. Ceramide and SM Hydrolyzing Activity of Purified SM Deacylase 

**Figure 7:**
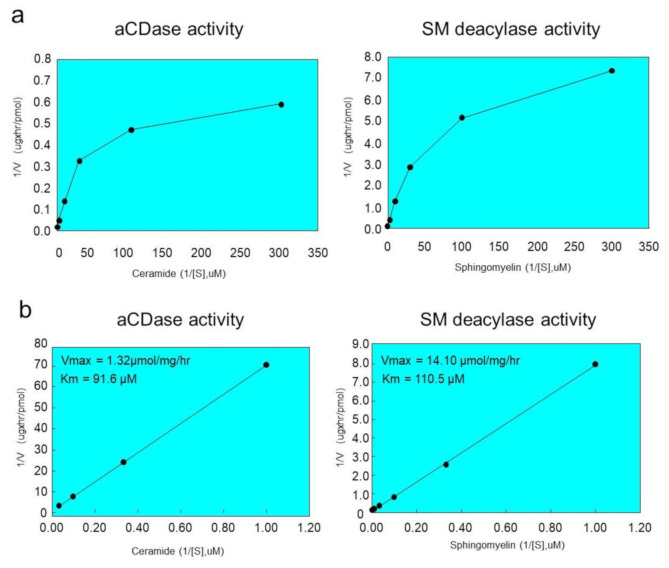
Kinetic analysis of purified rat SM deacylase activity and aCDase activity. (**a**) Purified enzyme was incubated for 12 h at 37 °C with varying amounts of SM or ceramide substrates. The final reaction mixtures contained 50 mM potassium acetate buffer (pH 4.7), enzyme source, substrate, 0.1% Triton X-100 and 20 mM CaCl_2_. The rate of SPC or sphingosine (SPH) generation as a function of SM deacylase or aCDase was measured, respectively. (**b**) Lineweaver–Burk plot (double reciprocal) for the reaction: 1/S (SM or ceramide concentration) versus 1/V (rate of SPC or SPH formation). The lines represent the data fit for the production of the corresponding products. Results are expressed as means from two separate experiments.

We next examined the enzymatic characteristics of SM deacylase activity purified from rat skin. The K_m_ and V_max_ values of SM deacylase for the hydrolysis from SM to SPC were 110.5 µM and 14.1 nmol/mg/h, respectively (Figure 7). We then asked whether purified SM deacylase could hydrolyze ceramide, since it is identical to the β-subunit of aCDase. The results showed the generation of sphingosine (SPH) with a specific activity of 91.6 µM and 1.32 nmol/mg/h at 37 °C (K_m_ and V_max_, respectively) (Figure 6). This result seems to contrast with our previous report [19], which demonstrated that SM deacylase partially purified as a pI 4.7 fraction from the SC of AD patients has no aCDase activity. It is probable that during the chromatography and/or IEF steps, an endogenous inhibitor that was tightly bound to SM deacylase was separated that resulted in the increased SM deacylase activity. This endogenous inhibitor molecule might affect the aCDase activity of the SM deacylase-active pI 4.7 fraction, and after separation during further purification, a relatively inactive catalytic site of aCDase may be opened to various molecules, including ceramide. 

## 3. Discussion

In the present study, SM deacylase from rat skin was purified to homogeneity with an apparent molecular mass of 43 kDa, an enrichment of >14,000-fold, and maximal pH and pI values of between 5.5 and 6.0 and around 8.0, respectively. The purified SM deacylase followed normal Michaelis–Menten kinetics with V_max_ and K_m_ of 14.1 nmol/mg/h and 110.5 µM, respectively. These properties of pH dependency and molecular weight (MW) are consistent with those (pH = 4.7, MW = 40 kDa) observed in our previous study of AD skin [19]. However, the pI value of SM deacylase was different from our previous report using analytical IEF of a homogenate of the SC of AD skin, which detected pI values of SM deacylase, GlcCDase, aSMase and aCDase of 4.2, 7.4, 7.0 and 5.7, respectively [19]. In this study, the enzymatic features of SM deacylase were definitely changed during the IEF, i.e., the pI value shifted from 5.0 in the skin homogenate to 8.0 in the purified enzyme with an enhancement of activity by ~200-fold after IEF (Table 1). Those data suggest that acidic inhibitory proteins or N-linked carbohydrate moieties are removed from the pI 4.2 protein complex during the purification. This supports the possible removal of N-linked carbohydrate moieties from the purified SM deacylase. Another contrasting enzymatic property includes the substrate specificity for ceramide in which the unpurified SM deacylase active fractions (pI 4.2 fraction) from the SC of AD skin did not exhibit any activity (as detected using ^14^C-labeled palymitoyl-SPH of aCDase [19]). In contrast, the purified SM deacylase in this study followed normal Michaelis–Menten kinetics for aCDase activity (the hydrolysis from ceramide to SPH) (as measured by the release of SPH using LC-MS-MS) with its K_m_ and V_max_ values of 91.6 µM and 1.32 nmol/mg/h, respectively. He et al. [31] already reported that their purified recombinant human aCDase protein has an acidic pH optimum and follows normal Michaelis–Menten kinetics with V_max_ and K_m_ of 27.8 µmol/mg/h and 389 µM, respectively. The very small V_max_ value of our purified SM deacylase for the enzymatic activity of aCDase indicates that our purified SM deacylase has a relatively weak aCDase activity compared with the previously reported one [31]. We thought it likely that the significantly lower than expected Vmax for acid ceramidase activity is due to removal of the α-subunit via unknown mechanisms. On the other hand, SM deacylase can also play a role as GCer deacylase to hydrolyze GCer to GS and free fatty acid [19] but determination of that activity is under investigation as further studies. Analysis by MALDI-TOF MS/MS using a protein spot with SM deacylase activity separated by 2D-SDS-PAGE allowed its amino acid sequence to be determined and then identified as the β-subunit of aCDase, which consists of α- and β-subunits linked by one disulfide bond (C31/C340). The identification of SM deacylase as the β-subunit of aCDase was also corroborated by the Western blotting of samples demonstrating that the purified SM deacylase was detectable with the β-subunit antibody at ~43 kDa in the reduced conditions, while recombinant human aCDase was detectable with the β-subunit antibody at ~43 kDa and at ~52kDa in the reduced and non-reduced conditions, respectively. Consistently, breaking the disulfide bond (C31/C340) of recombinant human aCDase by dithiothreitol (DTT) elicited the activity of SM deacylase with ~40 kDa upon gel chromatography. These results strongly support the conclusion that the purified SM deacylase is identical to the β-subunit of aCDase. aCDase catalyzes the hydrolysis of ceramides to yield SPH and fatty acid to regulate many cellular processes and had been purified to apparent homogeneity from urine [31] and placenta [33] whose full-length cDNA was determined by Koch et al. [34]. The protein is N-glycosylated with rat aCDase with four glycosylation sites, whereas human aCDase has an additional two sites [35,36,37]. Three specific glycosylation sites were shown to be required for autocleavage to occur [35] whose structure reveals a configuration where two of them form extensive bridging interactions between the α- and β-subunits in the proenzyme [35]. In the skin, aCDase exists especially in the epidermis, including the SC [5,19] and plays an important role in SPH-1-phosphate-related signaling in keratinocytes [38] as well as in the ceramide-degrading process in the SC [5]. Autoproteolytic cleavage and activation in human and in rat aCDases have been documented in which the formation of a hydrogen bond between Asp-162 and Cys-143 elicits a conformational change, allowing Arg-159 to act as a proton acceptor, which in turn results in facilitating an intermediate thioether bond between Cys-143 and Ile-142 (Met-142 in rats), the site of aCDase cleavage into α- and β-subunits, which is an essential requirement for the activation [36,39]. This was also corroborated by the fact that treatment of recombinant human aCDase with the cysteine protease inhibitor methylmethane thiosulfonate abrogated both the cleavage and the enzymatic activity [39]. As for the natural activation mechanisms of aCDase, Gebai et al. [36] speculated in their crystal aCDase model that the three-dimensional configuration of the substrate-binding channel in activated aCDase after autocleavage appears to be specific for ceramide, as acyl residue-containing sphingolipids with bulky head groups such as SM and GCer would result in steric hindrance and be unable to work as a substrate for aCDase [36]. As for the role of the S-S cross-linking (C31/C340) between the α- and β-subunits in functional aCDase activity, Gebai et al. [36] also reported that human and rat proenzyme aCDases contain six cysteines, four of which form two disulfide bonds. One disulfide bond stabilizes a turn in the β-subunit (C388/C392), whereas the other covalently latches the N-terminal end of the α-subunit linker to the β-subunit (C31/C340). Thus, the α- and β-subunits are intimately associated with burying a total of 894 Å^2^, maintaining a heterodimeric state even after autocleavage. Taken together with the significantly lower than expected Vmax of the purified SM deacylase for acid ceramidase activity, this evidence indicates that the α-subunit of aCDase is essentially required for accelerating the enzymatic reaction but not for expressing its activity.

**Figure 8:**
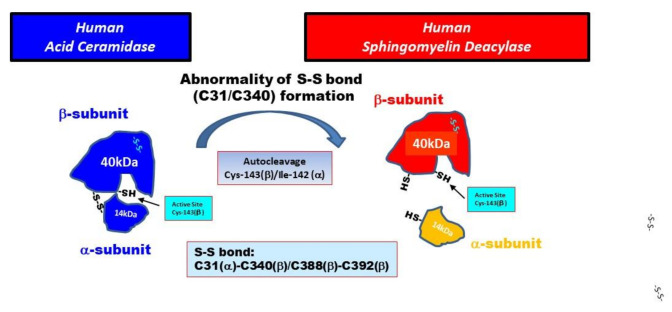
Hypothetical mechanisms involved in the expression of sphingomyelin deacylase in atopic dermatitis (AD) skin.

It is likely that in the epidermis of healthy skin, the proenzyme aCDase undergoes autocleavage into α- and β-subunits in the intracellular lysosomal system without breaking the S-S bond between the α- and β-subunits to acquire aCDase activity. Consistently, western blotting using antibodies to the α- and β-subunits reveals the aCDase protein with a 50 kDa molecular weight in the SC of healthy skin (data not shown). In contrast, in the epidermis of AD skin, as depicted in Figure 8, we thought it likely that the β-subunit would be generated both by auto-cleavage of the covalent peptide bond between Ile-142 in the α-subunit and Cys-143 in the β-subunit and by breaking the S-S bond (C31/C340) between the α- and β-subunits of aCDase via unknown mechanisms, which leads to the induction of SM deacylase activity. Therefore, it is intriguing to determine whether the β-subunit of aCDase can be detected at 40 kDa in AD skin under the non-reduced conditions, but this is still under investigation. In this regard, we have already reported that the detectable activity of aCDase occurs in healthy SC with a decreased level in AD SC, which is paralleled by the decreased level of ceramide [5,19], suggesting the possibility that the expression and activation of SM deacylase following both cleavages may contribute to the diminished activity of aCDase in the AD SC. Thus, it is probable that both cleavages result in deleting the hindrance in the enzymatic active pocket against acyl residue-containing sphingolipids with bulky head groups such as SM and GCer and leads to the expression of the activities of SM deacylase and possibly GCer deacylase, which occur as enzymatic deacylation reactions in the same active pocket as aCDase.

Based upon the above findings, we hypothesize two possible causative biological factors that underlie the expression of SM deacylase in AD skin as follows: (1) The formation of the S-S bond between the α- and β-subunits of aCDase could be impaired in AD skin, presumably due to a possible point mutation of the aCDase proenzyme, although no such point mutations are currently known to exist. (2) Breaking the S-S bond could occur more easily in AD skin than in healthy skin because of unknown mechanisms.

In conclusion, our finding that the pathogenic ceramide-degrading enzyme SM deacylase, discovered as a causative factor for down-regulating ceramide synthesis in the SC of AD skin, is identical to the β-subunit of aCDase provides an essential and deep insight into understanding the pathogenesis of AD. This should facilitate therapeutic approaches for possible specific inhibitors of SM deacylase that could be applied topically or orally to essentially abrogate the ceramide deficiency in AD skin, which would result in the essential cure of AD.

## 4. Materials and Methods

### 4.1. Materials and Antibodies

Pamitoyl SM and SPC were purchased from BIOMOL Research Laboratories (Plymouth Meeting, PA, USA), and 18-carbon chain ceramide was obtained from Avanti Polar Lipids (Alabaster, AL, USA). Methanol, chloroform of HPLC grade and n-octyl-β-d-glucoside were purchased from Nakarai Pharmacy (Kyoto, Japan). Ampholytes were purchased from Bio-Rad (Hercules, CA, USA). Other chemicals were from Sigma (St. Louis, MO, USA), unless otherwise specified. Horseradish peroxidase-conjugated secondary antibody against rabbit IgG was purchased from BD Bioscience (San Jose, CA, USA). The rabbit polyclonal anti-aCDase β-subunit antibody was prepared as follows: A polypeptide corresponding to Cys292-Glu306 of the human aCDase β-subunit was synthesized and injected into rabbits. The polyclonal antibodies were purified by conventional chromatography, as described previously, and were further subjected to affinity chromatography using a peptide-conjugated resin [16]. A single band corresponding to the expected molecular size for the aCDase β-subunit was observed in western blots of the purified antibodies for recombinant human aCDase. These results suggested that the antibodies are specifically reactive to the β-subunit of aCDase.

### 4.2. Assays for SM Deacylase and aCDase Activities

**Figure 9:**
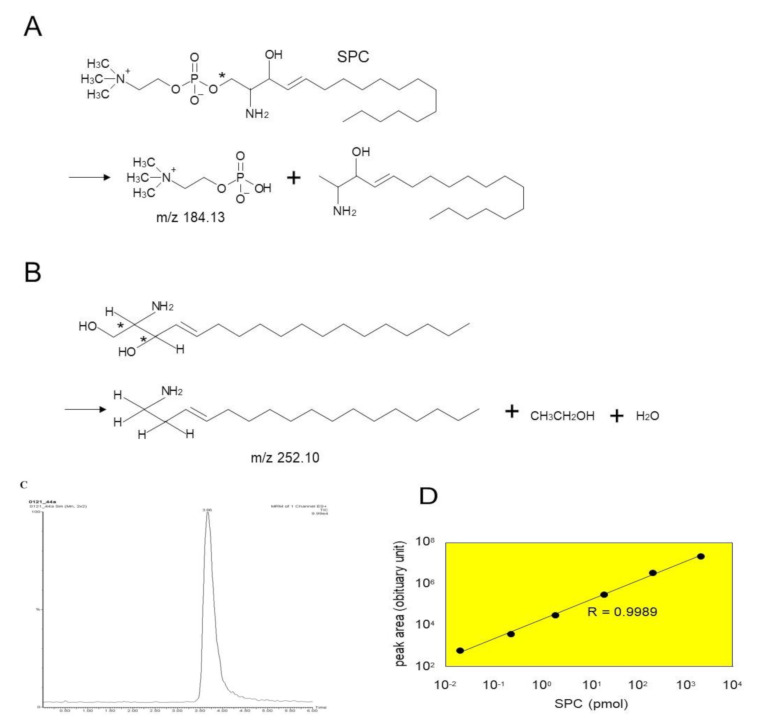
Quantification of the reaction product of SM deacylase (SPC) by LC-MS/MS. (**A**) Molecular structure of SPC and the predicted MS/MS fragments. SPC was fragmented into two parts at the site indicated by the asterisk. The fragment of *m*/*z* 184.13 was detected to calculate the amount of SPC. (**B**) Structure of SPH shown with asterisks that indicate collision sites by MS/MS. The fragment of *m*/*z* 252.10 was detected to calculate the amount of SPH. (**C**) LC-MS/MS chromatograms of the MS/MS fragment of SPC. SPC (20 pmol) was injected into the LC-MS/MS and analyzed according to the method described in the Materials and Methods. (**D**) Standard curve prepared with different amounts of SPC; the peak areas were plotted against 0.02–2000 µM SPC.

The enzyme reactions were carried out in the same conditions as reported previously [22]. The solubilized enzyme was incubated with 20 μM SM at 37 °C for 1–16 h in 50 mM potassium acetate (pH 4.7), 1% n-octyl-β-d-glucoside (*w*/*v*) and 20 mM CaCl_2_. The reactions were terminated by the addition of two volumes of chloroform:methanol (2:1, *v*/*v*). The reaction solutions were mixed vigorously and centrifuged for 10 min at 1000× *g*. The upper phase was applied to OASIS HLB (60 mg/3 cc, Waters, Beverly, MA, USA) and the resin was washed with 10% methanol. The reaction product (SPC) was eluted by methanol for quantitative analysis. The eluted samples were injected onto a Develosil ODS UG-3 column (2 mm i.d. × 10 cm, Nomura Chemical Inc., Tokyo, Japan) equilibrated in 5 mM ammonium acetate, 0.1% trifluoroacetic acid (TFA) and 80% methanol at a flow rate of 0.2 mL/min. The eluent was introduced into the ion spray source of a Micromass Ultima tandem mass spectrometer (Waters, Beverly, MA, USA) operated in the positive ion mode. Settings were as follows: capillary voltage = 3.7 kV, cone voltage = 35 V, source temperature = 70 °C, desolvation temperature = 200 °C, cone gas = 35 l/h, desolvation gas = 650 l/h, multiplier voltage = 700V. The analyte was detected with selected reaction monitoring (SRM) of the transition *m*/*z* 465.45 > 184.13 (Figure 9A). A sharp peak spectrum was observed for SPC in SRM mode, as shown in Figure 9C. Quantitation was accomplished using a standard curve ranging from 0.2 to 2000 nM, and its linearity was above 0.998 (Figure 9D) The aCDase activity assay was carried out under the same conditions as SM deacylase, except that the solubilized enzyme was incubated with 20 μM ceramide as a substrate. The reaction product (sphingosine, SPH) was detected with SRM of the transition *m*/*z* 300.5 > 252.1 (Figure 9B). Its linearity was almost the same as the SPC generation.

### 4.3. Purification of SM deacylase

#### 4.3.1. Preparation of Skin Extracts from Rat Skin

Dorsal skins of male Wistar rats (4 weeks old) were used as a source of SM deacylase enzyme. They were collected and homogenized in a buffer containing 50 mM potassium acetate, pH 4.7, 5% n-octyl-β-d-glucoside (*v*/*v*) and 20 mM CaCl_2_ followed by centrifugation at 10,000× *g* for 30 min at 4 °C. After adding ammonium sulfate to 30% saturation (*w*/*v*), the sediments were re-solubilized by adding lysis buffer with sonication. The supernatants were collected and filtered using a 0.22 μm filter unit. The supernatants were eluted through a Phenyl-5PW column (TOSOH, Tokyo, Japan) after equilibration in washing buffer-P (50 mM sodium acetate, pH 5.0, 2 M NaCl and 0.25% n-octyl-β-d-glucoside (*w*/*v*)). Washing steps were performed with two column volumes of washing buffer-P, and elution steps were performed by flowing three column volumes of elution buffer-P (50 mM sodium acetate, pH 5.0, 0.15 M NaCl and 1% n-octyl-β-d-glucoside (*w*/*v*)). Typical peaks were collected by measuring the absorbance at 280 nm.

#### 4.3.2. Purification of Skin Extracts

##### Isoelectric Focusing

The eluted fractions were diluted with electrophoresis buffer (2% (*v*/*v*) ampholytes (pH 3.0–10.0) and 5% n-octyl-β-d-glucoside (*w*/*v*)), and were applied to a preparative IEF chamber (Bio-Rad, Hercules, CA, USA), followed by resolving at 12W constant power for 5 h at 4 °C. SM deacylase active fractions were filtered through a 30 kDa cutoff filter (Millipore, Billerica, MA, USA), concentrated and collected in bottles.

##### Ion Exchange Chromatography

The solvent was loaded onto a 55 mL SP Sepharose HP (2.6 × 10 cm, 26/10, GE Healthcare, Little Chalfont, England) equilibrated with a 5 L bed volume of running buffer (50 mM potassium acetate, pH 4.7, and 1% n-octyl-β-d-glucoside (*w*/*v*)). Bound proteins were eluted from the column using a NaCl gradient (from 0 to 1000 mM). Active fractions were concentrated using a Centricon 100 (Amicon, Danvers, MA, USA) at 4700× *g* to a final volume of approximately 1 mL.

##### Gel Filtration Chromatography

The concentrated eluates were applied to a Superdex 200 column (GE Healthcare, Little Chalfont, England) equilibrated with buffer supplemented with 50 mM potassium acetate, 150 mM NaCl and 1% n-octyl-β-d-glucoside (*w*/*v*). Proteins were eluted at a flow rate of 1 mL/min and fractions corresponding to 45 kDa were collected. Aliquots of those fractions were analyzed for SM deacylase activity, and fractions containing high SM deacylase activity were pooled and concentrated to 1 mL using a 30 kDa ultrafree centrifugal column at 4 °C (Millipore, Billerica, MA, USA). Samples were further purified through a Superdex 200 column again in a similar way.

##### Chromatography with a C20 Column (Similar to Affinity Chromatography)

The concentrated gel filtration eluant was loaded onto an RP-415 C20 column (Shodex, Tokyo, Japan) that had been equilibrated with washing buffer-P (50 mM potassium acetate, 150 mM NaCl and 1% n-octyl-β-d-glucoside (*w*/*v*), pH 4.8). The washing and elution steps were carried out with washing buffer-P and elution buffer-P (50 mM potassium acetate, 150 mM NaCl and 5% n-octyl-β-d-glucoside (*w*/*v*), pH 4.8), respectively. The flow rate was maintained at 1 mL/min during the procedure. Fractions from the elution step that contained high SM deacylase activity were pooled, then concentrated to less than 1 mL using a 30 kDa ultrafiltration membrane (Millipore, Billerica, MA, USA).

### 4.4. In-gel Protein Digestion and Protein Identification by MALDI-TOF-MS

Protein spots were manually excised from 2D-PAGE gels, and digested according to the conventional method. The dried material, including digested peptides, was dissolved in 10 μL 0.1% TFA (*v*/*v*). To remove excess salts from the extracts, solid-phase extraction was performed using a ZipTip C18 (Millipore) according to the manufacturer’s instructions. Peptides were eluted from the ZipTip with 2.5 μL 50% ACN, 0.1% TFA and 1 μL of the eluants were spotted onto a target plate. A matrix solution containing 0.3 mg/mL α-cyano-hydroxycinnamic acid in 33% acetone and 66% ethanol was prepared, and 0.5 μL of that matrix solution was immediately mixed with the sample solution in the target well. MALDI-TOF MS measurements were performed using an ultraflex MALDI-TOF/TOF-MS (Bruker Daltonics, Bremen, Germany) operating in reflector mode with 25 kV accelerating voltage and 26.5 kV reflecting voltage. MS/MS analysis was performed in LIFT mode, using post source decay. The parameters were: accelerating voltage 8.0 kV, Lift1 voltage 19.0 kV, Lift2 voltage 2.2 kV, and Reflector voltage 29.0 kV. Peptide fragments obtained by MS/MS analysis were de novo sequenced supported by DataAnalysis version 3.0 software (Bruker Daltonics).

### 4.5. Expression and Preparation of Recombinant Human aCDase

A full-length human aCDase or aCDase lacking an inter-subunit disulfide bond (C31A) was inserted into a pcDNA3.1(+) expression vector. These expression vectors or pcDNA3.1(+) itself were transfected into HEK293 cells using Trans-it LT1 (Mirus; Madison, WI, USA). At 48 h post transfection, the medium was collected and mixed with SDS-PAGE sample buffer without reducing agent. aCDase (C31A) was separated into alpha- and beta-subunits by electrophoresis. For biochemical experiments, recombinant aCDase was prepared as follows: The medium containing expressed aCDase was subjected to affinity chromatography using ConA-sepharose (GE healthcare; Little Chalfont, England). The fraction containing aCDase was filtrated using an Ultrafree filtration unit (10,000 MW, Millipore; Billerica, MA, USA) with 10 mM Tris-HCl (pH 7.5) containing 20 mM NaCl.

## Figures and Tables

**Table 1:** Typical purification steps of SM deacylase from rat skin. SM deacylase activity was determined by measuring the production of SPC by LC-MS/MS as described in the Materials and Methods section.

Step	Total Protein	Total Activity	Specific Activity	Enrichment	Yield
	mg	pmol	pmol/mg	fold	%
homogenate	2447	6.3 × 10^4^	26	1	100
Phenyl-5PW	293	2.2 × 10^4^	76	3	35
Rotofor	55	8.8 × 10^5^	16089	622	1398
SP-Sepharose	27	7.5 × 10^5^	27735	1072	1183
Superdex 200	2	7.6 × 10^5^	363919	14067	1207
Shodex RP18-415	-	2.4 × 10^5^	-	-	379

**Table 2:**
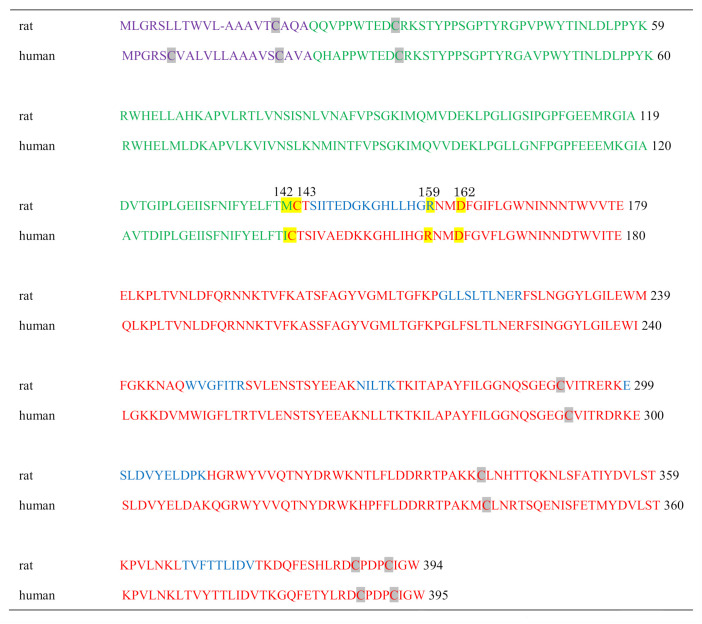
aCDase β-subunit hits by MASCOT database.

Purple: signal sequence; Green: alpha subunit; Red: beta subunit; Blue: detected by MS/MS.

## Abbreviations

AD	atopic dermatitis
aCDase	acid ceramidase
SM	sphingomyelin
SPC	sphingosylphosphorylcholine
PC	phosphorylcholine
SPH	sphingosine
IEF	isoelectric focusing
SC	stratum corneum
pCer	synthetic pseudo-ceramide
DTT	dithiothreitol
BGCase	β-glucocerebrosidase
aSMase	acid sphingomyelinase
LGs	lamellar granules
GCer	glucosylceramide
SPT	serine-palmitoyl transferase
SCD	stearoyl CoA desaturase
GCERS	GCer synthase
SMS	GCer synthase
GS	glucosylsphingosine
HCs	healthy controls
